# Lower gastrointestinal tract dysbiosis in persistent critical illness: a systematic review

**DOI:** 10.1099/jmm.0.001888

**Published:** 2024-10-09

**Authors:** Emily Tang, Nicholas Doan, Tess Evans, Edward Litton

**Affiliations:** 1 School of Medicine, University of Western Australia, Nedlands, Australia; 2 Intensive Care Unit, Royal Brisbane and Women’s Hospital, North Metropolitan Health Service, Brisbane, Australia; 3 University of Queensland Centre for Clinical Research, Herston, Australia; 4 Intensive Care Unit, Fiona Stanley Hospital, South Metropolitan Health Service, Perth, Australia

**Keywords:** critical illness, dysbiosis, gut, microbiome

## Abstract

**Introduction.** The human lower gastrointestinal tract microbiome is complex, dynamic and prone to disruption occurring during critical illness.

**Hypothesis or gap statement**. The characteristics of lower gastrointestinal tract microbiome disruption and its association with clinical outcomes in patients with prolonged intensive care stay remain uncertain.

**Aim**. To systematically review studies describing lower gastrointestinal tract molecular sequencing in patients with prolonged intensive care stay and explore associations with clinical outcomes.

**Methodology**. This systematic review was prospectively registered and follows the Preferred Reporting Items for Systematic Reviews and Meta-analyses guidelines. OVID MEDLINE, EMBASE and The Cochrane Central Register of Controlled Trials databases were searched for eligible studies describing adults and/or children who underwent molecular sequencing of stool or rectal samples taken on or after 10 days of intensive care.

**Results**. There were 13 studies with 177 patients included. The overall certainty of evidence was low, and no studies reported mortality. Reduced alpha diversity was observed in nine out of nine studies but was not associated with clinical outcomes in four out of four studies. Longitudinal alpha diversity decreased in five out of six studies, and inter-individual beta diversity increased in five out of five studies. After approximately one week of intensive care unit admission, rapid fluctuations in dominant taxa stabilized with trajectories of either recovery or deterioration in five studies. Pathogenic enrichment and commensal depletion were reported in all 13 studies and associated with clinical outcomes in two studies.

**Conclusion**. Lower gastrointestinal tract microbiome disruption is highly prevalent and has consistent characteristics in patients with prolonged intensive care stay. Amongst reported metrics, only relative taxon abundance was associated with clinical outcomes.

## Introduction

The human lower gut microbiome is composed of a dynamic community of bacteria, fungi, viruses and archaea. Dysbiosis, an alteration of the microbiota characterized by loss of diversity, overgrowth of pathogenic species and loss of commensal organisms [1], is prevalent in patients admitted to the intensive care unit (ICU).

Dysbiosis in critical illness is thought to be associated with poorer clinical outcomes though there have been inconsistent findings across microbiome indices [2]. Population methods of evaluating microbiota composition include alpha diversity, beta diversity and relative taxon abundance. Alpha diversity, most commonly calculated by Shannon or Simpson’s indices [2], captures the richness (number of taxa) and evenness (uniformity of taxon population size) *within* a sample. Beta diversity measures diversity *between* samples, which can reveal differences between body sites, longitudinally within an individual, and between individuals. In a systematic review by Evans *et al*. [2], reduced alpha diversity during the first 72 h of ICU admission was not associated with in-hospital mortality. Rather, the relative abundance of taxa such as *Enterococcus* spp. offered stronger prognostic capability [2].

This review focussed on persistent critical illness (PCI), defined as patients remaining in intensive care due to ongoing illness when the reason for admission is no longer predictive of outcomes [3]. This occurs from approximately day 10 of ICU admission based on epidemiological studies [4,7]. Patients with PCI are at a high risk of adverse outcomes and pose a therapeutic challenge with a disproportionate economic burden on ICU services [4]. PCI exposures, such as sepsis, and the burden of therapeutic interventions in nosocomial infections, total parenteral nutrition, medications and mechanical ventilation, are each expected to exacerbate dysbiosis [8]. However, the characteristics of the lower gastrointestinal microbiota in PCI remain uncertain.

The aim of this review was to characterize lower gastrointestinal tract dysbiosis amongst PCI patients and describe its associations with clinical outcomes. We hypothesized that in patients with PCI, there would be consistent, characteristic changes in the lower gastrointestinal microbiome and that enrichment of pathogenic taxa (such as *Enterococcus* spp.) would be associated with increased hospital mortality.

## Methods

### Search study and selection criteria

This systematic review was conducted in accordance with the Preferred Reporting Items for Systematic Reviews and Meta-analyses (PRISMA) guidelines [9] and prospectively registered on PROSPERO (CRD42023404047).

Studies were eligible for inclusion if they described a cohort of adults and/or children admitted to an ICU, who underwent molecular sequencing (including 16S rRNA and shotgun metagenomic sequencing), with reporting of one microbiome or clinical outcome. To examine PCI, studies needed to have at least one stool or rectal sample taken on or after day 10 of ICU admission. Studies were excluded if they were not peer-reviewed, abstract-only, culture-based only, non-English language publications or if participants were neonates.

### Outcomes

The primary outcomes related to the microbiome were alpha diversity, beta diversity and relative taxon abundance. The primary clinical outcome was all-cause hospital mortality. Secondary clinical outcomes included all-cause mortality at the longest follow-up, ICU length of stay, hospital length of stay, quality of life indices, nosocomial infection and multi-drug-resistant (MDR) organism colonization.

Pre-specified subgroups included adults compared to paediatrics, sepsis compared to non-sepsis, nosocomial infection compared to without nosocomial infection, ICU setting (medical, surgical, trauma, burns, neurosurgical, cardiothoracic and liver), higher illness severity compared to lower illness severity, rectal sample compared to stool sample, antibiotics compared to no antibiotics prior to sampling and enteral nutrition compared to parenteral nutrition.

### Study selection

The electronic databases OVID MEDLINE, EMBASE and The Cochrane Central Register of Controlled Trials were searched for relevant articles (Supplementary Appendix, available in the online version of this article), followed by manual reference searching of all eligible studies. The search was limited to articles published after 2012, considering the recent application of molecular sequencing techniques. Two reviewers (E.T. and N.D.) independently screened search results with a third reviewer adjudicating discrepancies (T.E.). Corresponding authors were contacted where data were required to clarify eligibility and/or outcomes.

### Data extraction and synthesis

Two reviewers (E.T. and N.D.) independently extracted data followed by cross-checking. Data were entered into a pre-piloted standardized data collection form. Risk of bias assessment was performed according to the Risk of Bias Assessment Tool for Nonrandomized Studies (RoBANS) [10], a tool designed to be applicable for various observational study designs.

### Data analysis

Meta-analysis was planned if data were available from at least three studies with comparable exposure and outcome variables. The standardized mean difference would be calculated to compare continuous data, whilst categorical outcomes would be measured through an odds ratio. The planned meta-analysis involved binary transformation of microbiome indices, to compare clinical outcomes between patients with high versus low dysbiosis, using a random-effects model with heterogeneity estimated by I^2^ statistic. A *P* value of <0.05 would be considered significant. Sensitivity analysis was planned excluding studies with a high risk of bias.

## Results

**Fig. 1.**
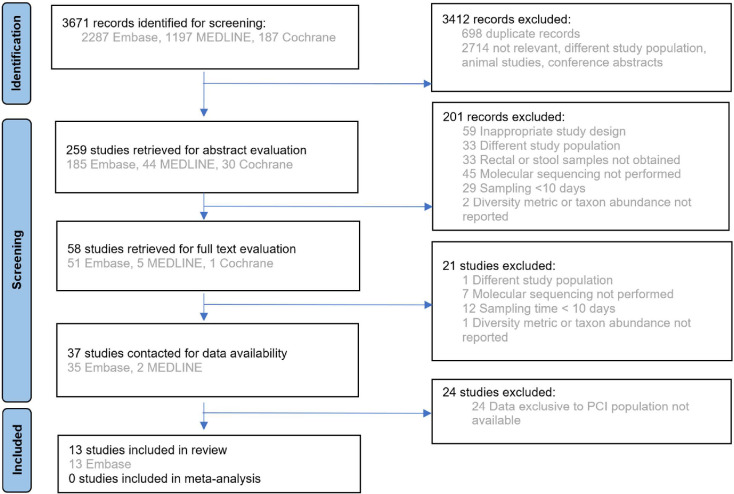
PRISMA flowchart[9].

**Fig. 2.**
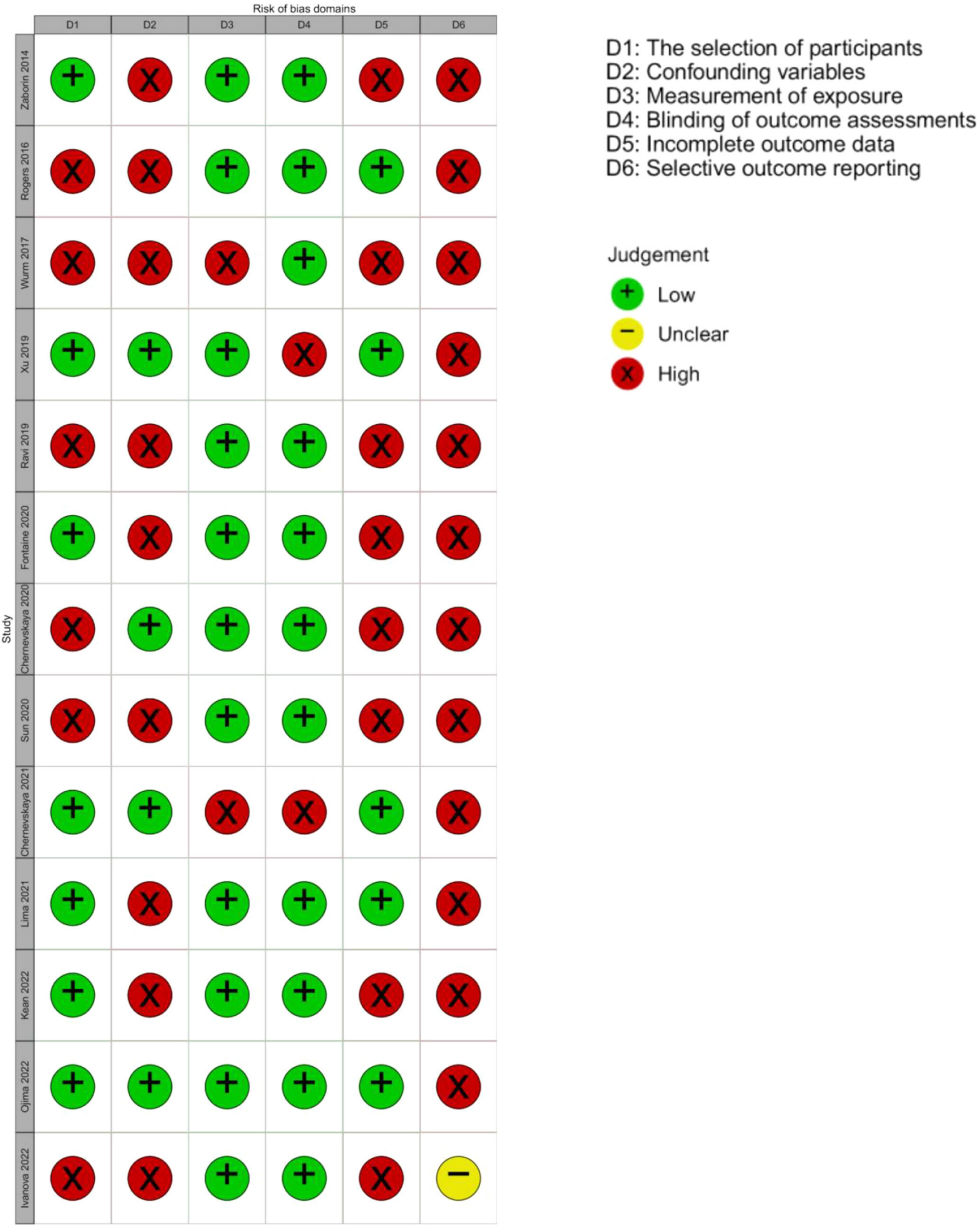
RoBANS included in this review [10].

There were 3671 records screened, and 13 studies with 177 participants (166 adults and 11 children), published between September 2014 and June 2022, were included in the systematic review. The PRISMA flowchart is displayed in Fig. 1, and a description of included studies is provided in Table 1. Alpha diversity was reported in 12 studies, with eight reporting the Shannon index and three reporting Simpson, Chao1 richness or operational taxonomic unit number. Beta diversity was reported in five studies. Relative taxon abundance was reported in all studies. Methodological differences between studies have been summarized (Table S1, available in the online version of this article). Samples were collected at least once weekly in 10 out of 13 studies from admission until ICU discharge, with timepoints up to day 100 of ICU admission. The risk of bias, as displayed in Fig. 2, was high for the domains of selection of participants, confounding variables, incomplete outcome data and selective outcome reporting.

**Table 1.** Characteristics of cohorts included in the systematic review

First author	Year of publication	Country	No. of participants	No. of PCI participants (%)	No. and type of centres			Sampling	Subspeciality or diagnostic group	Comparison group
Zaborin	2014	USA	14	10 (71.4)	1	Mixed*	Adult	Longitudinal	General	Healthy controls‡
Rogers	2016	USA	37	9 (24.3)	1	Mixed	Paediatric	Cross-sectional	General	Healthy controls§
Wurm	2017	Austria	3	2 (66.7)	1	Mixed	AdultPaediatric	Longitudinal	Apoptotic enterocolitis	No controls
Xu	2019	China	98	35 (35.7)	1	Medical	Adult	Longitudinal	Neurological	Healthy controls§
Ravi	2019	England	24	24 (100)	1	Mixed	Adult	Longitudinal	General	No controls
Fontaine	2020	France	31	7 (22.5)	1	Medical	Adult	Longitudinal	General	No controls
Chernevskaya	2020	Russia	18	7 (38.9)	2	Surgical	Adult	Longitudinal	Neurosurgical	Healthy controls‡Acute critical illness‡
Sun	2020	China	15	15 (100)	1	Medical	Adult	Longitudinal	AECOPD**	No controls
Chernevskaya	2021	Russia	44	44 (100)	1	Medical	Adult	Longitudinal	Neurological	Healthy controls§
Lima	2021	USA	10	8 (80)	1	Medical	Adult	Longitudinal	Burns	Healthy controls‡Burn nurses‡
Kean	2022	UK	70	1 (1.4)	3	Mixed	Paediatric	Longitudinal	General	Healthy controls§
Ojima	2022	Japan	71	13 (18.3)	1	Mixed	Adult	Longitudinal	General	Healthy controls‡
Ivanova	2022	Russia	2	2 (100)	1	Mixed	Adult	Longitudinal	Neurological	No controls

*Medical and surgical patients.

**†Acute Eexacerbation of Cchronic Oobstructive Ppulmonary Ddisease.

+ ‡Unmatched.

++§Matched.

The planned meta-analysis was not carried out due to the lack of available primary outcome data, study heterogeneity in excess of the prespecified threshold and few mortality events. Results have been presented qualitatively in Table 2.

**Table 2.** Association between diversity or pathogen enrichment and clinical outcomes amongst included studies

First author	Year of publication	No. of PCI participants	Alpha diversity	Pathogen enrichmentRelative abundance (highest % amongst PCI participants)	Clinical outcomes and associations	
					Alpha diversity associations	Pathogen associations
Zaborin	2014	10	Low	*Enterococcus* spp. (99.9%)*Staphylococcus* spp. (99.4%)Family *Enterobacteriaceae* (99.9%)Family *Pseudomonadaceae* (30%)Phylum *Proteobacteria*	Not reported	Not reported
Rogers	2016	9	Low	*Enterococcus* spp. (90.6%)	Not reported	Not reported
Wurm	2017	2	Low	Family *Enterobacteriaceae* (97.5%)Phylum *Proteobacteria* (99.9%)	Not reported	Not reported
Xu	2019	35	Low	Family *Enterococcaceae* (>70%)Family *Enterobacteriaceae* (>70%)	Not reported	Not reported
Ravi	2019	24	Low	*Enterococcus* spp. (99%)*Escherichia coli* (80%)*Candida albicans* (75.6%)	SOFA*: Not associated with alpha diversity	Not reported
Fontaine	2020	7	Not recorded	*Enterococcus* spp.	*MDR-GNB*†: Not associated with alpha diversity	Not reported
Chernevskaya	2020	7	Low	*Enterococcus* spp.*Streptococcus* spp.*Staphylococcus* spp.Family *Enterobacteriaceae*Family *ErysipelotrichaceaeRuminococcus gnavus*	Not reported	Not reported
Sun	2020	15	Not recorded	Phylum *Proteobacteria* (15.6%)	SOFA*: Not associated with alpha diversity	Not reported
Chernevskaya	2021	44	Not recorded	*Eggerthella lentaEnterococcus* spp.Family *Enterobacteriaceae*	NIHSS‡, Rankin scale and Rivermead Mobility Index: Not associated with alpha diversity	*NIHSS*‡: Family *Clostridiaceae* negatively associated; Family *Alcaligenaceae and* Family *Prevotellaceae* positively associated*Rivermead Mobility Index:* Family *Clostridiaceae*/*Lachnospiraceae* and *Ruminococcus* spp. positively associated*Rankin scale:* Family Clostridiaceae/Lachnospiraceae, *Ruminococcus* spp.*, Coprococcus* spp*. and Roseburia* spp*.* negatively associated
Lima	2021	8	Low	*Prevotella* spp. (12.9%)Phylum *Proteobacteria* (13%)	Not reported	Not reported
Kean	2022	1	Low	Family *Enterococcaceae*Family *Enterobacteriaceae*Family *Streptococcaceae*Family *Staphylococcaceae*	Not reported	Not reported
Ojima	2022	13	Low	*Enterococcus* spp.Phylum *Proteobacteria* (12.7%)	Not reported	SOFA*: Increased SOFA* at admission and rise in SOFA* during admission both associated with *Actinobacteria* spp.
Ivanova	2022	2	Not recorded	*Enterocytozoon bieneusi* (91%)*Candida glabrataAspergillus* spp.Phylum *Proteobacteria*	Not reported	Not reported

*Sequential Organ Failure Assessment score.

**†Multi-drug-resistant Gram-negative- bacteria.

***‡National Institute of Health Stroke Score.

### Alpha diversity

Out of 12 studies reporting alpha diversity, nine made comparisons to healthy controls with all finding reduced alpha diversity in PCI [11,19].

Diverging longitudinal changes in alpha diversity were observed. In five out of six studies, alpha diversity decreased progressively with increasing length of ICU stay [12,14, 18,20]. However, recovery in alpha diversity by time of ICU discharge was observed in some patients [12,14, 16, 20].

### Beta diversity

Inter-individual comparison between PCI patients showed progressive divergence in beta diversity over time, as noted in all five reporting studies [12,14, 17,19]. Interestingly, increasing differences between PCI and healthy control patients were no longer observed beyond day seven of ICU admission [17,19].

Intra-individual variability in PCI patients, characterized by rapid fluctuation of dominant taxa between bi-weekly samples, was observed in all five evaluating studies [11,13,19, 21]. This was observed at the phylum level [11,19] and was responsive to administration and cessation of probiotics [13].

Loss of site specificity was also observed [12].

### Pathogenic enrichment

Pathogen enrichment was highly prevalent in all 13 studies [11,23], as displayed in Table 2. PCI was associated with phylum enrichment of *Proteobacteria* and disproportionate reductions in typically dominant *Bacteroidetes* and *Firmicutes* in five studies [11,13, 17, 19, 22]. The relative abundance of *Proteobacteria* was as high as 99.9% in certain patients [13]. After approximately a week of ICU admission, there may be a stabilization of microbiota composition [19,20], including *Bacteroidetes*, *Firmicutes* and *Proteobacteria* phyla abundance, regardless of the antibiotic group administered [19].

At the genus level, pathogen dominance greater than 90% was observed for *Enterococcus* spp., [11,12, 14, 15] *Staphylococcus* spp. [11,12, 16] and the *Enterobacteriaceae* family [11,15, 16]. Rogers *et al.* [12] reported a longitudinal increase in the relative abundance of dominant taxa during the ICU stay accompanied by an increase in the relative abundance of *Enterococcus* spp*.* [19] and *Enterobacteriaceae* family [14]. An increase in the relative abundance of *Enterococcus* spp. was associated with a reduction in alpha diversity [23].

Whole genome sequencing (WGS) revealed fungal enrichment of *Candida* spp.*,* comprising up to 75% of certain microbiomes, *Aspergillus* spp*.* and *Enterocytozoon bieneusi* [15,22]. The abundance of *E. bieneusi*, a cause of human microsporidiosis, which presents with diarrhoea and wasting in the immunocompromised, was as high as 91% of total fungal abundance [22].

### Commensal abundance

Depletion of commensal organisms, such as *Ruminococcus* spp., *Faecalibacterium* spp.*, Roseburia* spp., *Blautia* spp. and *Coprococcus* spp*.* [12,16, 18, 21], was consistently observed with further longitudinal decreases in abundance [14,15, 18, 19]. Specific commensal bacteria and the archaea *Methanobrevibacter smithii* withstood ICU environmental conditions and increased in relative abundance to >50% [15]. Chernevskaya *et al.* [16] observed an increased relative abundance of commensals *Erysipelotrichaceae* spp*.* and *Ruminococcus gnavus*, which may have potential as opportunists in the vulnerable host, after two weeks of ICU admission compared to early admission.

### Subgroup analysis

A total of five studies reported the effect of antibiotics on the lower gut microbiome in PCI patients. Carbapenems were associated with reduced alpha diversity [15,21], pathogen dominance [15] and drastic reductions of the phylum *Actinobacteria* [19]. The number of antibiotic classes that were used pre-sampling was associated with a loss of commensals [21], and combinations of six antibiotics were associated with MDR bacteria development [11].

There were insufficient data to report subgroups of adults versus paediatrics, sepsis versus non-sepsis, nosocomial infection versus without nosocomial infection, ICU setting, higher illness severity versus lower illness severity, rectal versus stool sample and enteral nutrition versus parenteral nutrition.

### Clinical outcomes

Where assessed, alpha diversity was not prognostic of clinical outcomes [15,20, 21, 23]. All three studies [15,20, 21], which observed the relationship between alpha diversity and disease severity, found no association. Reduction in alpha diversity was not associated with MDR Gram-negative bacteria colonization [23].

Relative taxon abundance was associated with disease severity. Ojima *et al.* [19] found that disease severity was positively associated with the phylum *Actinobacteria*. Chernevskaya *et al.* [21] identified associations between relative taxon abundance and neurological outcomes. Poorer neurological outcomes were associated with *Alcaligenaceae* and *Prevotellaceae* families. Meanwhile, retention of commensals such as the *Clostridiaceae*/*Lachnospiraceae* family, *Ruminococcus bromii*, *Coprococcus* spp. and *Roseburia* spp*.* was correlated with improved neurological outcomes.

None of the studies examined relative taxon abundance and mortality during our time frame. Two studies included in the analysis assessed the prognostic potential of the microbiome in the first week of ICU admission, finding that increased abundance of the family *Enterobacteriaceae* [14] and an imbalanced *Bacteroidetes:Firmicutes* (B:F) ratio of >8 or <1/8 (19) were associated with mortality.

There were insufficient data to report on ICU length of stay, hospital length of stay, quality of life indices and nosocomial infection.

## Discussion

In this systematic review that included 13 studies and 177 participants, patients with PCI appear to demonstrate consistent characteristics of lower gastrointestinal tract dysbiosis. Fall in alpha diversity, increased beta diversity, pathogenic taxa enrichment and commensal loss were reported. Intra-individual fluctuations in dominant taxa are prominent, suggesting reduced ecological stability that may be responsive to time-dependent interventions including antibiotic and probiotic administration.

This systematic review supports previous findings in acute critical illness that relative taxon abundance may provide more relevant information to clinical practice than traditional ecological measures of alpha diversity. Studies conducted in general and neurological ICUs found promising associations between relative taxon abundance in PCI and clinical features [14,19, 21]. Most of the available literature on critical illness has focused on early ICU admission, and it appears that the marked changes in the first week of admission modulate the subsequent trajectory of the microbiome in PCI and mortality [14,17, 19]. This may have meaningful implications for the timing of prognostic marker utilization, microbiome-based interventions and antibiotic stewardship.

PCI patients exhibited major changes to their microbiome structure over time, deserving attention. Interestingly, the time frame for PCI beginning at day 10 of ICU admission appears to align with a pivotal point for the lower gastrointestinal microbiome at approximately one week of ICU admission when rapid fluctuations in dominant taxa are observed to stabilize and the microbiome ceases to diverge from healthy controls [17,19, 20]. From this point of severely reduced ecological stability, microbiome trajectories of either recovery or deterioration were observed (Fig. S1, available in the online version of this article) with parallels to clinical recovery or progression of PCI. This time frame is also consistent with an increase in incident antimicrobial resistance development in the ICU [24,25], which may influence the diverging microbiome trajectories.

Longitudinal changes in the lower gut microbiome were not readily predictable, and the mechanisms underlying different microbiome trajectories require further study. The severity of dysbiosis increased with the length of ICU stay in five studies [12,14, 18,20] and may be due to cumulative adverse exposures associated with ICU admission. However, some patients with PCI experience recovery in microbiota diversity whilst still in ICU [12,14, 16, 20]. This observation may illustrate differences in microbiome trajectory between single and multiple organ dysfunction corresponding to a prior understanding of dysbiosis as a driver of multiple organ dysfunction [26]. It has been proposed that diagnosis [18,19] and disease severity [12] can also account for differing trajectories, although there is a paucity of evidence and reproducibility. The gross disparity between the small PCI cohorts included in the studies compared to very large biorepositories containing samples from healthy participants is an important constraint. Modulators were not interrogated in this systematic review due to a lack of available data.

The clinical implications of the mycobiome and cross-kingdom interactions, particularly *Candida*–bacteria interactions, are an area of evolving research [27], although not captured in studies using 16S rRNA. Both studies employing WGS enabled the characterization of archaea and fungi, which evidenced fungal dominance warranting dedicated exploration at scale. Novel approaches, such as Hi-C metagenomics, which combines shotgun metagenomics with chromosome conformation capture, afford finer information on microbiome structure and evaluate virulence and antimicrobial resistance dynamics by examining bacteria, plasmid and bacteriophage links [22].

This systematic review reinforces the importance of antibiotic rationalization, given that significant ecological disruption was associated with an increased number of antibiotic classes and carbapenems specifically.

Characteristics of the metabolome, including serum and faecal metabolites, were evaluated in four of the included studies [13,16, 18, 21]. Whilst outside the scope of this review, metabolomics represents an area of prognostic potential, especially when integrated with microbiome characterization.

### Limitations

The number of studies and overall sample size are small, and the certainty of evidence is low. There were inconsistencies in the conduct of sampling, pre-processing, sequencing, post-processing and reporting.

Associations between dysbiosis and clinical outcomes were seldom reported, and there were insufficient data to interrogate the pre-specified secondary outcomes, modulators and subgroups.

Heterogeneity at each step of the metagenomic pipeline poses a significant challenge for the synthesis of microbiome studies (Fig. S2, available in the online version of this article). Standardized protocols are not available, and in their absence, it is important for studies to present a robust pre-publication statistical analysis plan with a rationale for the methods used. The Strengthening The Organization and Reporting of Microbiome Studies (STORMS) guideline represents significant progress towards comprehensive microbiome reporting [28], although it was not referred to in the 13 studies included in this systematic review.

### Future directions

**Fig. 3.**
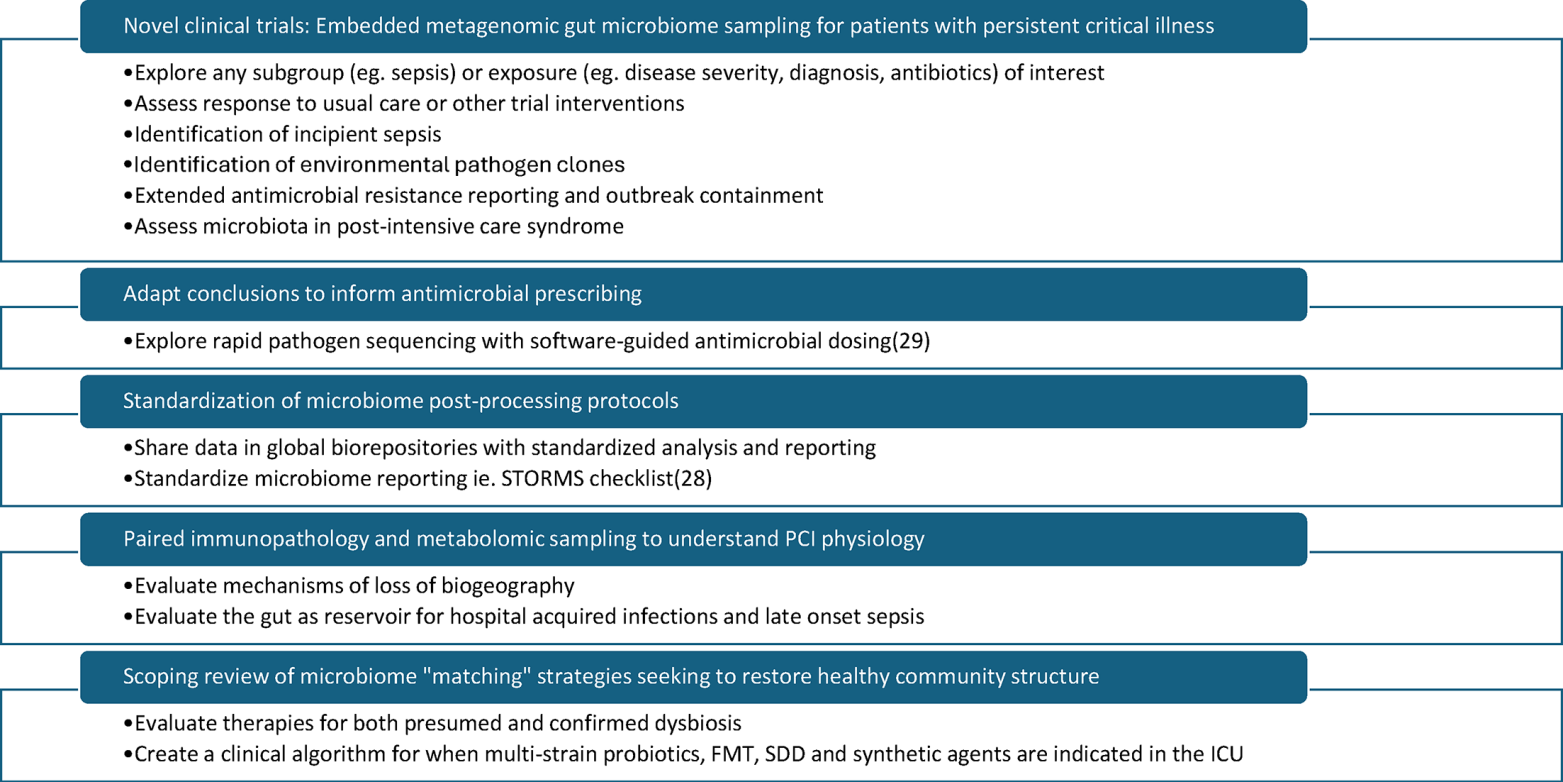
Future directions. FMT, faecal microbiota transplant; SDD, selective digestive decontamination [28,29].

There is an urgent need for robust, adequately powered, longitudinal clinical studies that assess the prognostic capability of relative taxon abundance, the effect of modulators and important cohorts such as patients with sepsis and PCI (Fig. 3).

## Conclusion

Lower gastrointestinal tract microbiome disruption is highly prevalent and has consistent characteristics in patients with prolonged intensive care stay. Amongst reported metrics, only relative taxon abundance was associated with clinical outcomes.

## Highlights

- Lower gut dysbiosis is highly prevalent in patients with prolonged ICU stay, consistently characterized by reduced diversity, pathogenic enrichment, commensal depletion and dramatic fluctuation in dominant taxa.
- Relative taxon abundance may be of greatest clinical utility.
- After approximately one week of ICU admission, rapid fluctuations in dominant taxa stabilized with trajectories of either recovery or deterioration.
- Standardization of metagenomic approaches is required for meaningful synthesis of microbiome studies.

## supplementary material

10.1099/jmm.0.001888 Uncited Supplementary Material 1.

## Abbreviations

ICU: intensive care unit
MDR: multi-drug-resistant
OTU: operational taxonomic unit
PCI: persistent critical illness
PRISMA: Preferred Reporting Items for Systematic Reviews and Meta-analyses
RoBANS: Risk of Bias Assessment Tool for Nonrandomized Studies
STORMS: Strengthening The Organization and Reporting of Microbiome Studies
WGS: whole genome sequencing
